# Exploring Antimicrobial Peptides Efficacy against Fire Blight (*Erwinia amylovora*)

**DOI:** 10.3390/plants12010113

**Published:** 2022-12-26

**Authors:** Miloud Sabri, Kaoutar El Handi, Franco Valentini, Angelo De Stradis, El Hassan Achbani, Rachid Benkirane, Toufic Elbeaino

**Affiliations:** 1 Productions Végétales, Animales et Agro-Industrie, Faculté des Sciences, Ibn Tofail University, Kenitra 14000, Morocco; 2 Laboratory of Phyto-Bacteriology and Biocontrol, Plant Protection Unit-National Institute of Agronomic Research INRA, Meknès 50000, Morocco; 3 Istituto Agronomico Mediterraneo di Bari (CIHEAM-IAMB), Via Ceglie 9, 70010 Valenzano, BA, Italy; 4 National Research Council of Italy (CNR), Institute for Sustainable Plant Protection (IPSP), University of Bari, Via Amendola 165/A, 70126 Bari, BA, Italy

**Keywords:** *E. amylovora*, AMPs, lytic, non-lytic, ecofriendly control

## Abstract

Antimicrobial peptides (AMPs) are a various group of molecules found in a wide range of organisms and act as a defense mechanism against different kinds of infectious pathogens (bacteria, viruses, and fungi, etc.). This study explored the antibacterial activity of nine candidates reported in the literature for their effect on human and animal bacteria, (i.e., *Escherichia coli, Staphylococcus aureus*, and *Pseudomonas aeruginosa*) against *Erwinia amylovora* (*E. amylovora*), the causal agent of fire blight disease on pome fruits. The antibacterial activity of these peptides against *E. amylovora* was evaluated in vitro using viable-quantitative PCR (v-qPCR), fluorescence microscopy (FM), optical density (OD), and transmission electron microscopy (TEM), while the in vivo control efficacy was evaluated in treating experimental fire blight on pear fruits. With a view to their safe and ecofriendly field use in the future, the study also used animal and plant eukaryotic cells to evaluate the possible toxicity of these AMPs. Results in vitro showed that KL29 was the most potent peptide in inhibiting *E. amylovora* cell proliferation. In addition, the results of v-qPCR, FM, and TEM showed that KL29 has a bifunctional mechanism of action (lytic and non-lytic) when used at different concentrations against *E. amylovora*. KL29 reduced fire blight symptoms by 85% when applied experimentally in vivo. Furthermore, it had no impact on animal or plant cells, thus demonstrating its potential for safe use as an antibacterial agent. This study sheds light on a new and potent antibacterial peptide for *E. amylovora* and its modes of action, which could be exploited to develop sustainable treatments for fire blight.

## 1. Introduction

*Erwinia amylovora* (*E. amylovora*) is a highly destructive plant pathogenic bacterium causing fire blight disease, which affects species of the *Rosaceae* family. This disease has a devastating impact on apple (*Malus domestica* Borkh.), pear (*Pyrus communis* L.), and quince (*Cydonia oblonga* Mill.), making it a major concern for pome fruit producers worldwide [1]. The bacterium can infect all aerial parts of the host, causing wilting and death of shoots, twigs, and flower clusters, in addition to trunk blight, limb blight, and fruit and leaf blight [2]. It spreads rapidly through the tree and can destroy an entire orchard within a single season [3].

To date, the main challenge of fire blight is due to the lack of effective phytosanitary measures to stop its spread and prevent enormous losses in pome fruit production [4]. Current methods are mainly based on preventive measures and applications of antibiotics, namely streptomycin and oxytetracycline [5]. However, the dramatic increase in antibiotic resistance has led many regions (e.g., the European Union) to ban their use [6]. Moreover, no other approved substance has the same effectiveness as streptomycin [4]. In recent years, alternative sustainable control solutions for fire blight have been proposed, such as bacteriophages [7], essential oils [8], antagonistic bacteria [9], and antimicrobial peptides (AMPs) [1].

In the last 10 years, the use of AMPs has attracted interest as a promising approach to plant disease control due to their high antibacterial activity, low toxicity to the host plant, and the slower emergence of resistance to them [10]. In general, AMPs kill bacteria through interaction with the membrane that leads to its permeabilization, or by interacting with intracellular targets (i.e., DNA, RNA, or ribosomes) without causing membrane permeabilization; some AMPs have been reported to use both mechanisms [11]. In addition, AMPs are components of the innate immune system of diverse organisms, such as insects, plants, and animals, and are not persistent compounds, which makes them suitable for the control of phytopathogenic bacteria, replacing or complementing the existing agrochemicals for sustainable agriculture [1]. Several AMPs that have proven effective against different human, animal, and plant pathogenic bacteria, i.e., *Escherichia Coli, Staphylococcus aureus*, *Pseudomonas aeruginosa*, *Xylella fastidiosa*, etc. [12,13,14,15], can have extended antimicrobial activities that have never been exploited against other bacterial pathogens. Therefore, the present work aimed to investigate for the first time the effectiveness of nine AMPs against different bacteria, with a view to their potential for use against fire blight.

## 2. Results and Discussion

### 2.1. Screening of Potential AMPs against E. amylovora

The spot tests for the nine AMPs revealed different levels of *E. amylovora* susceptibility to each peptide. Results showed that *E. amylovora* was susceptible to all the tested peptides from at 1000, 500 and 250 µM (Supplementary Table S1). However, only four peptides (GF19, FG22, IL14, and KL29) could kill *E. amylovora* at 50 µM. The four potential peptides that exhibited high antibacterial activity against *E. amylovora* were selected for further evaluation.

### 2.2. Effect of Selected AMPs on E. amylovora

**Figure 1:**
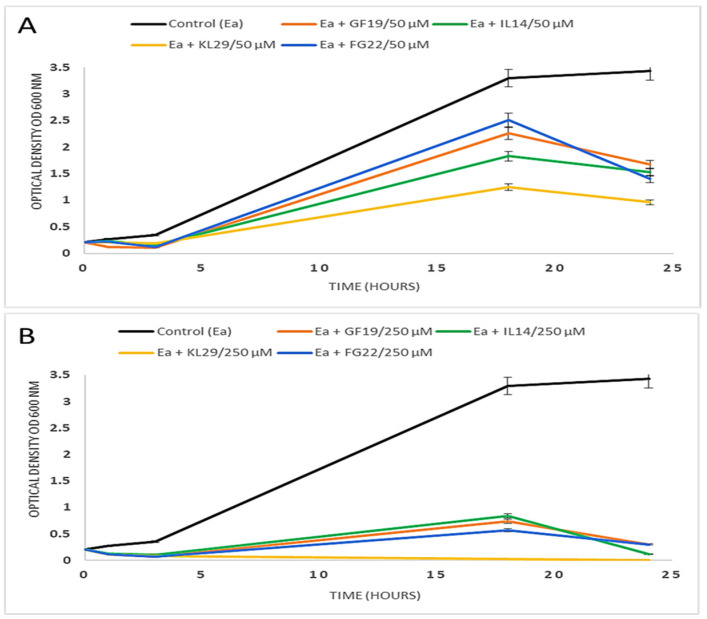
Optical density graphs showing the growth of *Erwinia amylovora*-treated antimicrobial peptides at 50 µM (**A**) and 250 µM (**B**). Error bars represent standard deviations of three replicates.

The turbidity experiment clearly showed the strong antibacterial activity of the selected AMPs against *E. amylovora*. At 50 µM, GF19, IL14, KL29, and FG22 inhibited *E. amylovora* growth by approximately 52%, 56%, 73%, and 60%, respectively (Figure 1). At 250 µM, both GF19 and FG22 inhibited *E. amylovora* growth by 92%, and IL14 by 97%, while KL29 inhibited all bacterial cell growth. In any case, *E. amylovora* growth was greatly affected when peptides were used at 250 µM, showing a direct relationship with peptide concentration. The different levels of *E. amylovra* growth inhibition is most likely due to differences in the physico-chemical properties of the tested AMPs, such as peptide length, amino acid constituents, the presence of positively charged residues, hydrophobicity, helicity of the spatial structure, and net charge of the molecule.

### 2.3. Bactericidal Activity of Selected AMPs

**Figure 2:**
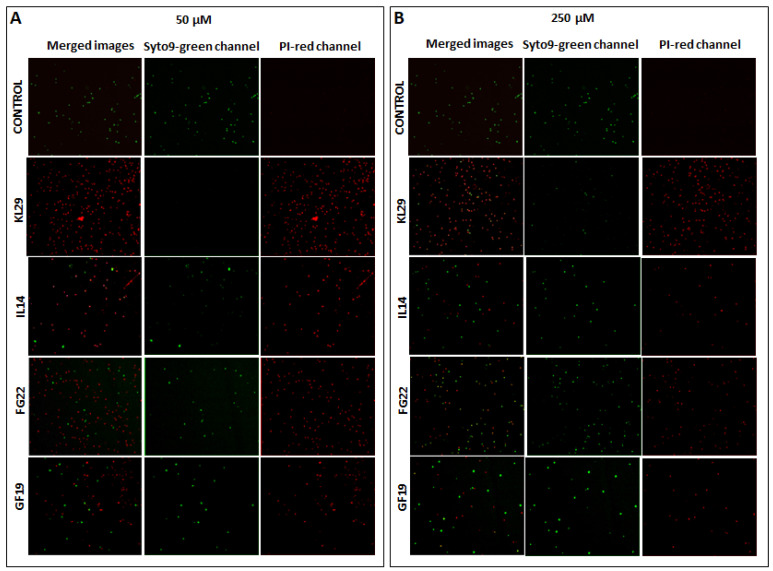
Fluorescence microscopy micrographs of untreated (control) and treated *Erwinia amylovora* cells with selected peptides at two concentrations (**A**) and (**B**) at 1 h. Viable and unviable cells are stained by SYTO9 (green fluorescence), while damaged dead cells were stained by propidium iodide, PI (red fluorescence). Magnification 40 X. The quantification of viable and unviable cells is reported in the Supplementary Table S2.

**Figure 3:**
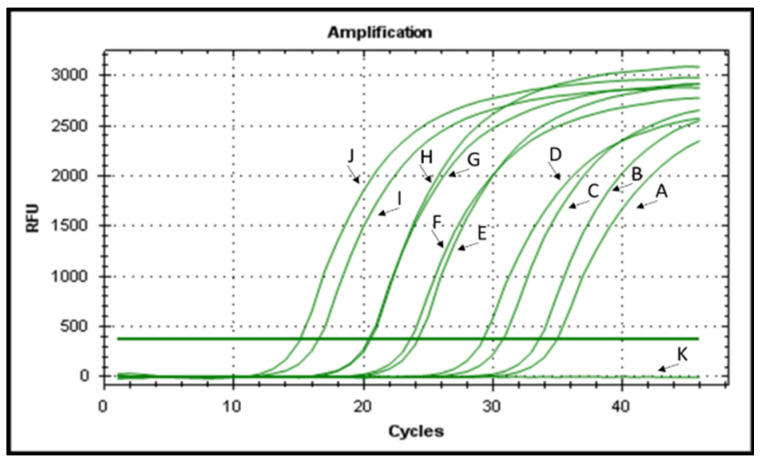
Viable-quantitative PCR DNA amplification curves obtained from (A) treated-*Erwinia amylovora* with KL29 at 50 µM; (B) IL14 at 50 µM; (C) KL29 at 250 µM; (D) IL14 at 250 µM; (E) FG22 at 50 µM; (F) GF19 at 50 µM; (G) FG22 at 250; (H) GF19 at 250 µM; (I) untreated *Erwinia amylovora*; (J) genomic DNA of *Erwinia amylovora* used as positive control; and (K) genomic DNA of *Xanthomonas campestris* used as a negative control. The v-PCR assay was performed in 4 replicates for each concentration. The result of a replication is displayed.

**Figure 4:**
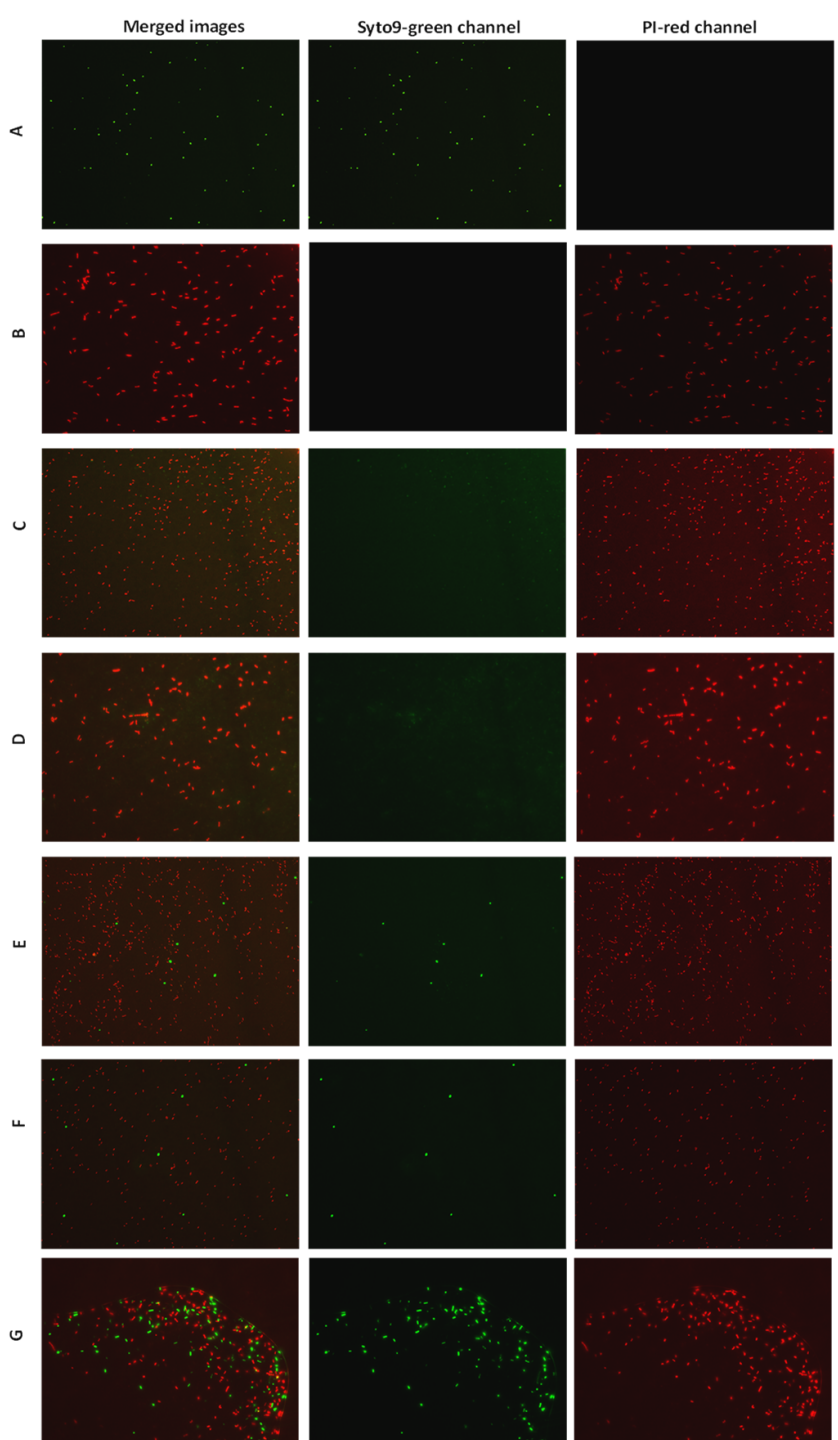
Fluorescence microscopy micrographs showing the effect of KL29 on *Xanthomonas campestris* pv. *campestris* cells treated with different concentrations: (**B**) 100 µM; (**C**) 50 µM; (**D**) 25 µM; (**E**) 12.5 µM; (**F**) 6.25 µM; (**G**) 2 µM. (**A**) untreated cells. Magnification 40X.

The bactericidal activity of the selected AMPs against *E. amylovora* was evaluated using v-qPCR and FM, both able to assess the integrity of *E. amylovora* cells. The FM micrographs showed green fluorescence for the untreated *E. amylovora* cells, indicating intact cell structures. However, peptide treatments led to a shift in fluorescence from green to red, indicating the loss of membrane integrity (Figure 2). There was no green fluorescence in the field of *E. amylovora* cells treated with KL29 at 50 µM. However, green cells were observed with KL29 treatment at 250 µM, suggesting that KL29 exhibits high lytic activity against *E. amylovora* at low rather than high concentration. This effect was observed with all the tested peptides; high concentration decreased peptide lytic activity against *E. amylovora*. This result was also confirmed by v-qPCR assay (Figure 3), where low peptide concentration was more efficient in lysis of *E. amylovora*. When these results are taken together with those of turbidity assay, it appears most likely that the tested AMPs exhibit two distinct mechanisms for killing *E. amylovora*: lytic at low concentration by interacting with cell membranes, and non-lytic at high concentration by traversing the cell membrane and blocking essential cellular processes without causing extensive membrane damage. Unlike *E. amylovora*, FM results showed that KL29-treated *X. campestris* was subjected to greater lytic activity at high peptide concentrations, while this activity diminished at low concentrations (Figure 4), showing a direct relationship to peptide concentration. This demonstrates that the ability of the tested peptides to kill bacteria by lytic or non-lytic activity varies according to the differences in composition of the bacterial membrane and in peptide concentration.

### 2.4. Lytic and Non-Lytic Activity of KL29

**Figure 5:**
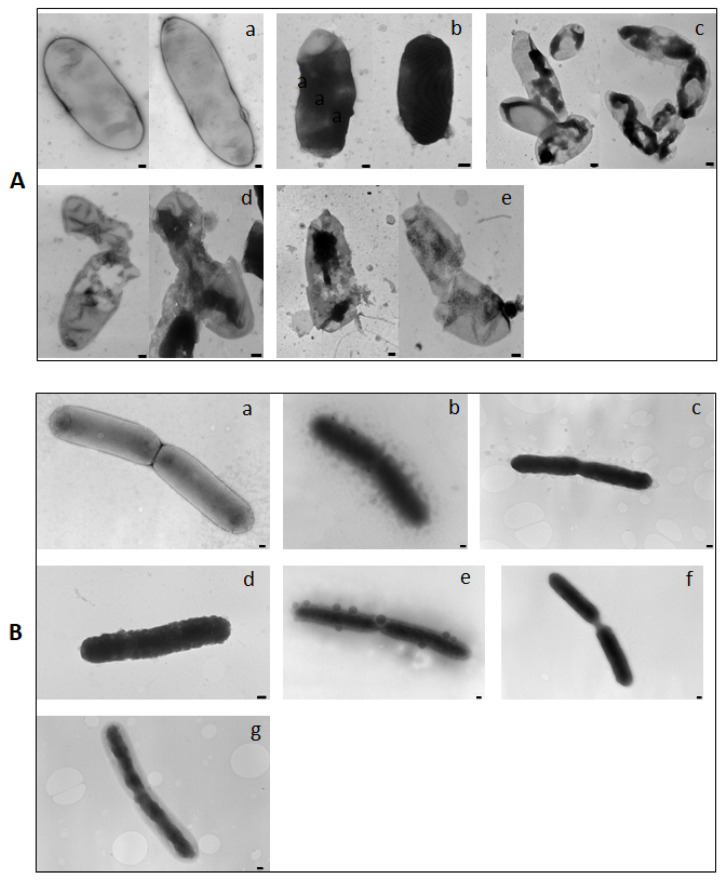
Transmission electron microscopy micrographs showing the bacteriostatic and bactericidal activities of KL29 against *Erwinia amylovora* (**A**) and *Xanthomonas campestris* pv. *campestris* (**B**). (**A**): (a) untreated cells of *Erwinia amylovora*; (b) *Erwinia amylovora* cells treated with KL29 at 250 µM; (c) with streptomycin; (d) with KL29 at 50 µM; (e) with ampicillin. (**B**): (a) untreated cells of *Xanthomonas campestris* pv. *campestris*; *Xanthomonas campestris* pv. *campestris* cells treated with different concentrations of KL29 at (b) 100 µM; (c) 50 µM; (d) 25 µM; (e) 12.5 µM; (f) 6.25 µM; (g) 2 µM. Bar = 100 nm.

TEM was performed on KL29-treated *E. amylovora* to further study the differential morphological cellular alterations caused by KL29 activity at low (50 µM) and high (250 µM) concentrations. Micrographs of *E. amylovora* cells treated with KL29 at 50 µM, and of those treated with 0.5 mg/mL of ampicillin, presented damaged cell walls and the decomposition of inner structures. Affected cells presented pore formation, cytoplasmic outflow, and wall fragmentation (Figure 5), which are typical features of membranolytic activity. KL29 is an amphipathic cationic peptide, and as such can interact with lipid components of bacterial membranes, which causes pore formation on cell membranes, and consequently leads to cell lysis. However, TEM micrographs of *E. amylovora* treated with KL29 at the high concentration showed low permeabilization of the outer membrane, cytoplasmic condensation, and loss of interior appearance (Figure 5). This was taken as evidence of the non-lytic antibacterial activity of KL29 on *E. amylovora,* at the high concentration. Additionally, cells treated with streptomycin (a bacteriostatic antibiotic that acts on the bacterial 30S ribosomal subunit) also presented reduced cell lysis and morphological alterations similar to those treated with KL29 at 250 µM, thus supporting the elevated non-lytic action mode of KL29 at the high concentration. Based on these findings, we assume that KL29 has two different antibacterial mechanisms of action against *E*. *amylovora*, which are regulated by the ability of *E. amylovora* to block the passage of peptide into the intermembrane space at low concentrations; therefore, KL29 at a low concentration interacts with the *E*. *amylovora* membrane and permeabilizes it, leading to complete cell lysis. Conversely, at high concentrations, KL29 traverses the cell membrane without causing its permeabilization, and binds to intracellular molecular targets (i.e., DNA, RNA, or proteins), consequently inhibiting the metabolic processes in *E. amylovora* bacteria, thus leading to cell death. Moreover, unlike conventional antibiotics, which interrupt cell growth and metabolic reaction by targeting specific protein receptors in bacteria, the tested AMPs proved to be bifunctional peptides with membranolytic and non-lytic killing properties. Therefore, it is difficult for bacterial cells to develop resistance to these because a range of genetic mutations would be required to compensate the damaged components.

### 2.5. Selected AMPs Innocuous for Eukaryotic Cells

**Figure 6:**
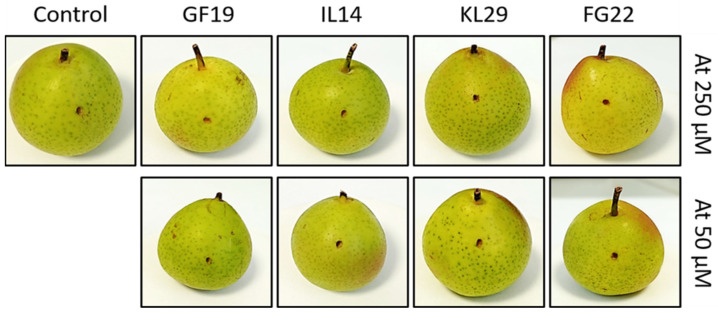
Effect of selected antimicrobial peptides on immature pear fruits, 5 days post injection with sterile water (control), GF19, IL14, KL29, and FG22 at 50 and 250 µM. Six replicates for each concentration and each peptide were used in this experiment.

**Figure 7:**
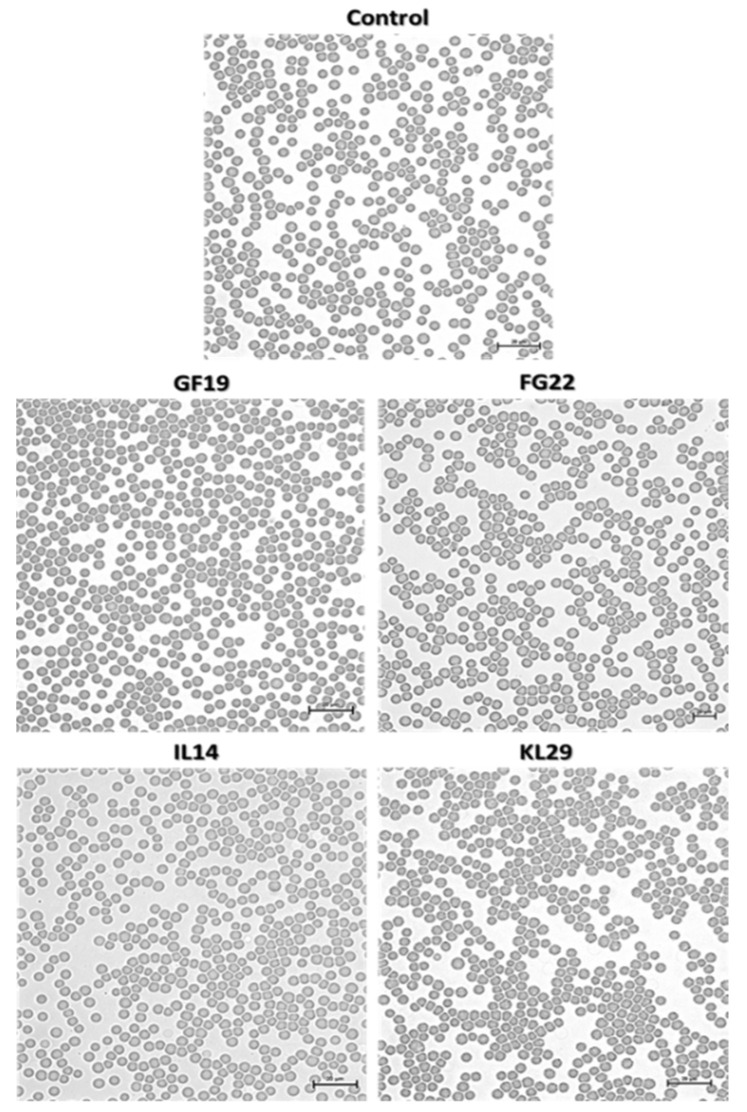
Horse erythrocytes challenged to selected antimicrobial peptides at 250 µM for 3 h, showing normal cells structure. Control: Untreated-erythrocyte cells. Bar = 20 µm.

The study of toxicity models is a hurdle in the development of antimicrobial peptides towards applications. In general, these models need very complex evaluation procedures, besides of being long term experiments, exhaustive, accompanied with permissions and license, etc., and often are not conclusive and always arguable. In this study, we tried to investigate the AMPs toxicity on pear fruits, and on a reductive (non-standard) model such as the exposure of erythrocyte cells from horse blood to the four selected AMPs. The results showed that none of the tested AMPs had hypersensitive or cytotoxic activities on horse erythrocytes or pears (Figure 6 and Figure 7). This outcome, showing that the tested AMPs are innocuous towards the plant cells and, despite the model used such as horse erythrocyte cells, are harmless, is an encouraging result that needs to be further reconfirmed by other models before being experimented in the field to contrast fire blight in infected trees.

### 2.6. In Vivo Antibacterial Activity of Selected AMPs against E. amylovora Infection

**Figure 8:**
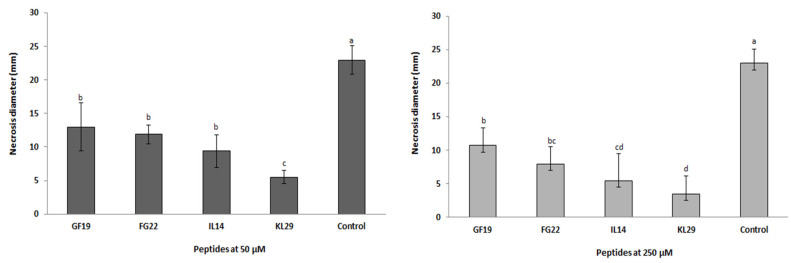
Effect of selected antimicrobial peptides on necrotic lesion diameter induced by *Erwinia amylovora* infection in immature pear fruits. The same letter indicates no statistical difference at *p* < 0.05. Error bars represent standard deviations of eight replications.

**Figure 9:**
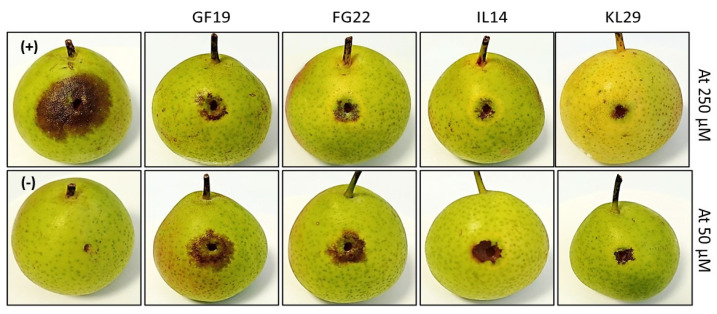
Antagonistic effect of selected antimicrobial peptides against *Erwinia amylovora* infection on detached immature pear fruits. (−) pear injected with sterile water and used as a negative control reaction; (+) pear fruit inoculated with *Erwinia amylovora* and used as a positive control reaction.

Statistical analysis conducted on AMP-treated immature pears infected with *E*. *amylovora* and on control fruits gave a *p* value < 0.001, showing the potential activity of AMPs (Figure 8). The intensity of fire blight symptoms (necrosis) that developed on *E*. *amylovora-*infected pears was significantly greater than on AMP-treated fruits (Figure 9). It is worth mentioning that symptom intensity varied according to the type of peptide used and to its concentration. KL29 was found to have the strongest antibacterial activity, reducing *E. amylovora* symptoms by 85% when used at 250 µM. Furthermore, KL29 reduced symptoms by 74% when used at 50 µM, demonstrating a direct relationship of its activity to the peptide concentration. Similarly, IL14 reduced disease symptoms by 61% at a concentration of 50 µM and by 76% at 250 µM, while FG22 reduced symptoms by 48% at 50 µM and by 65% at 250 µM. However, GF19 reduced symptoms by 43% at 50 µM and by 48% at 250 µM. These results were in line with the turbidity assay, which also confirmed the dual mechanisms of action (lytic and non-lytic) of the tested AMPs against *E*. *amylovora*. Interestingly, in addition to having bifunctional killing mechanisms and the strongest antibacterial activity against *E*. *amylovora*, KL29 has also been demonstrated to act as a defense elicitor by activating the plant immune system [16]. Hence, KL29 can be considered as an extremely promising antimicrobial agent for the development of ecofriendly and effective treatment for fire blight disease.

## 3. Materials and Methods

### 3.1. AMP Synthesis and Bacterial Strains

The nine AMPs, reported in Table 1, to be tested against *E. amylovora* were selected based on their strong antibacterial activities against human, plant, and animal bacteria, i.e., *Escherichia Coli*, *Staphylococcus aureus*, and *Xylella fastidiosa*, as described in the literature. All AMPs were synthesized by ProteoGenix (Schiltigheim, France) and their purity was assessed (>95%) by high-performance liquid chromatography (Table 1). Lyophilized peptides were dissolved in sterile water to a stock concentration of 1 mM and filter sterilized through a 0.22-μm-pore-size filter. The stock solution was diluted to obtain the desired final concentrations (500, 250, 100, 50, 25 and 12.5 μM).

The bacterial material consisted of *E. amylovora* strain PGL Z1, isolated in 2013 from pear in Apulia region (Italy), and *Xanthomonas campestris* pv. *campestris* strain CFBP 1710 (*X. campestris*), isolated in 1975 from *Brassica oleracea* var. *botrytis* in France, which was used as a control to investigate the bactericidal activity and action mode of AMPs against two different bacteria. The bacterial strains were cultured on yeast extract peptone glucose agar (YPGA) and broth (YPG) media, consisting of 0.5% yeast extract, 0.5% peptone, 1% glucose and 1.5% agar. *E. amylovora* and *X. campestris* cell suspensions were prepared in sterile distilled water and the suspensions were adjusted to an optical density at 600 nm (OD600) of 0.1, which corresponds approximately to 10^8^ CFU/mL.

### 3.2. Screening of Potential AMPs against E. amylovora

The nine AMPs were screened using the spot test to select those with the best antibacterial activity against *E. amylovora*. Briefly, 300 µL of *E. amylovora* suspension (10^8^ CFU/mL) was poured into the plates and maintained at room temperature for 15 min. Subsequently, 5 μL from each peptide at different concentrations (1000, 500, 250, 100, 50, 25 and 12.5 μM) was spotted onto the surface of the plates, followed by overnight incubation at 25 °C. Sterile distilled water was spotted in the plates as a control.

### 3.3. Turbidity Assay

The antibacterial activity of the selected peptides (GF19, FG22, IL14, and KL29) against *E. amylovora* was assessed by a test contact of 24 h coupled with optical density (OD600) measurements. Briefly, 20 µL from each concentration (50 and 250 µM) was mixed with 180 µL of *E. amylovora* suspension (YPG) and incubated at 25 °C for 24 h. Six OD600 nm measures were taken over 24 h using the NanoDrop™ One/OneC Microvolume UV-Vis Spectrophotometer (ThermoFisher Scientific, Waltham, MA, USA).

### 3.4. Viable-Quantitative PCR (v-qPCR) and Fluorescence Microscopy (FM)

The bactericidal activity of the selected peptides (GF19, FG22, IL14, and KL29) was assessed by a test contact for 24 h, as described (Section 2.3), coupled with v-qPCR assay using the PMAxx™ (Biotium, Rome, Italy). PMAxx™ is a photo-reactive dye that binds to DNA with high affinity; upon cell lysis, it covalently attaches to disrupted DNA that cannot be amplified by PCR. This unique feature makes PMAxx™ extremely useful in selective detection of bacterial cells with intact plasma membranes by qPCR. Briefly, after 24 h at 25 °C, 200 µL of the mixture was transferred to a new tube and treated with PMAxx at a final concentration of 7.5 μM. Subsequently, samples were incubated in the dark at room temperature for 8 min, followed by photoactivation for 15 min. Genomic DNA of all samples was extracted using the cetyltrimethylammonium bromide (CTAB) method [17]. v-qPCR was carried out in a thermocycler apparatus (Bio-Rad CFX96, BioRad, Milan, Italy), using Ea-lsc primers and conditions reported in Laforest et al. [18]. The v-qPCR cycles consisted of an initial denaturation step of 95 °C for 5 min; 45 cycles of 95 °C for 30 s and 60 °C for 30 s with fluorescence readings at each cycle. For the FM assay, aliquots of *E. amylovora* suspension were incubated with 50 and 250 μM of selected peptides for 1 h at room temperature. The same protocol was used for *X. campestris* at different concentrations (100, 50, 25, 12.5, 6.25, 2 μM) using KL29 peptide, known for its potent bactericidal activity against *X. campestris* [19]. The LIVE/DEAD ^®^BacLight™ viability kit (ThermoFisher Scientific, Milan, Italy) was used according to the manufacturer’s recommendation to assess the integrity of bacteria cells treated with the peptides at 1 h post-infection (Stocks, 2004). Briefly, 9 μL of sample was mixed with 0.5 μL of SYTO9 and 0.5 μL of propidium iodide (PI) for 15 min at room temperature in the dark. Photomicrographs were taken with a Nikon E800 microscope using a fluorescein isothiocyanate (480/30 excitation filter, DM505 dichroic mirror, 535/40 emission filter) and tetramethyl rhodamine isocyanate (546/10 excitation filter, DM575 dichroic mirror, 590 emission filter) fluorescence filter sets.

### 3.5. Transmission Electron Microscopy (TEM)

Aliquots of *E. amylovora* suspensions were incubated with the KL29 for 15 min at room temperature to investigate peptide antibacterial modes of action (lytic and non-lytic). *E. amylovora* suspensions treated with 0.5 mg/mL of streptomycin (bacteriostatic antibiotic), and ampicillin (bactericidal antibiotic) were used as controls. Preparations were observed under TEM (FEI MORGAGNI 282D, Hillsboro, OR, USA) by the dip method: the carbon coated copper/rhodium grids were incubated with treated and untreated bacterial suspension for 2 min, and then rinsed with 200 μL of distilled water. Negative staining was obtained by floating the grids on 200 μL of 0.5 % *w*/*v* UA-zero EM STAIN (Agar-Scientific Ltd., Stansted, UK) solution and observing them under the EM using an accelerating voltage of 80 kV.

### 3.6. Evaluation of AMPs Toxicity

The possible toxicity of the selected peptides was evaluated using immature pear fruits (*cv*. ‘*Recchia falsa*’), and horse erythrocyte cells, respectively, as plant and animal cell models. Briefly, six fruits for each peptide were pricked to approximately 5 mm deep and inoculated with 100 µL of the corresponding peptide at 50 and 250 µM concentrations. Fruits were kept in a chamber at room temperature for 5 days and repeatedly examined for the development of lesions. The peptide toxicity was measured as lesion diameter. Erythrocytes from a horse blood sample (provided by the University of Bari, Department of Veterinary Medicine, Valenzano, Italy) were used for the hemolytic assay. The erythrocytes were challenged with AMPs at 50 and 250 µM for 3 h and their possible hemolytic activity was observed under light microscope.

### 3.7. In Vivo Antibacterial Activity of AMPs

To evaluate the in vivo antibacterial activity of selected AMPs, immature pear fruits (*cv*. ‘*Recchia falsa*’) were inoculated with *E. amylovora* and AMPs were applied. Briefly, ten-week-old pears were disinfected by immersion in a 70% ethanol solution for 2 min and then rinsed 3 times in sterile distilled water. Disinfected fruits were pricked to approximately 5 mm deep, with a sterile micropipette tip and inoculated with 20 µL of *E. amylovora* suspension (10^8^ CFU/mL). Once the fruits had absorbed the bacteria, two concentrations of peptides (50 and 250 µM) were used; 20 µL of the concentration was introduced into the inoculation site and incubated in a humid chamber at 25 °C for 6 days. Fruits inoculated with sterile water and with *E. amylovora* suspension were included as controls. The experiment consisted of eight replicates per treatment, and symptoms were recorded at 6 days post-infection. The symptoms developed in immature pears (i.e., necrosis or bacterial exudates) were evaluated by measuring the diameter of necrotic lesions. The data were then analyzed using variance analysis (ANOVA), followed by Duncan’s multiple range test (α = 0.05), using SPSS statistical software (version 24.0, IBM, Armonk, N.Y., USA). All data were presented as mean values ± standard deviation (SD).

## 4. Conclusions

This study tested for the first time the effect of nine AMPs on *E. amylovora* infection and provides further understanding of the antibacterial modes of action of AMPs, which were found to inhibit *E. amylovora* both in vitro and in vivo. The selected AMPs (KL29, GF19, FG22, and IL14) were found to have lytic and non-lytic mechanisms of action capable of significantly inhibiting *E. amylovora* growth, and accordingly reducing fire blight symptoms on pear fruits. Furthermore, they are toxicologically safe for plant and animal cells, as was explored to a limited extent in the present study, which offers excellent prospects for the future exploitation of these AMPs in the control of fire blight in the field. Finally, it is fundamentally important to acquire better knowledge of AMP modes of action and to design optimized AMPs to avoid repeating the mistakes that have led to the current crisis of antibiotic resistance.

## Figures and Tables

**Table 1:** The sequence and origin of the antimicrobial peptides used in this work.

Peptide	Code	Sequence	Origin	References
Ascaphin-8	GF19	GFKDLLKGAAKALVKTVLF-NH2	Frog	[12]
DASamP1	FF13	FFGKVLKLIRKIF-NH2	Synthetic	[12]
DASamP2	IL14	IKWKKLLRAAKRIL-NH2	Synthetic	[12]
Lycotoxin I	IL25	IWLTALKFLGKHAAKHLAKQQLSKL	Spider	[12]
Maculatin 1.3	GF21	GLLGLLGSVVSHVVPAIVGHF-NH2	Frog	[12]
Piscidin 1	FG22	FFHHIFRGIVHVGKTIHRLVTG	Fish	[12]
1036	VK13	VQFRIRVRIVIRK-NH2	Synthetic	[13]
BP178	KL29	KKLFKKILKYL-AGPA-GIGKFLHSAK-KDEL-OH	Synthetic	[14]
RIJK2	RV12	RIVWVRIRRWFV-NH2	Synthetic	[15]

## Data Availability

No additonal data supporting reported results are produced.

## Supplementary Materials

The following supporting information can be downloaded at: https://www.mdpi.com/article/10.3390/plants12010113/s1, Table S1: Results of the spot tests for the nine antimicrobial peptides used against *Erwinia amylovora*; Table S2: The statistical analysis consisted of counting the coloured spots (green: SYTO9 and red: PI) in the different channels of 10 replicas for the two concentrations of each antimicrobial peptide and the relative percentages of viable cells is calculated. The histogram shows the % of cells inhibition.
