# Supplementary material for: Exploring Antimicrobial Peptides Efficacy against Fire Blight (Erwinia amylovora)
Source: Plants (Basel). 2022 Dec 26;12(1):113. doi: 10.3390/plants12010113 (PMC9824012; doi:10.3390/plants12010113)

Supplementary Table S1. Results of the spot tests for the nine antimicrobial peptides used against *Erwinia amylovora*.

Peptide	Code	1 mM	500 µM	250 µM	100 µM	50 µM	25 µM	12.5 µM
Ascaphin-8	GF-19	+	+	+	+	+	-	-
DASamP1	FF-13	+	+	+	-	-	-	-
DASamP2	IL-14	+	+	+	+	+	-	-
Lycotoxin I	IL-25	+	+	+	+	-	-	-
Maculatin 1.3	GF-21	+	+	+	-	-	-	-
Piscidin 1	FG-22	+	+	+	+	+	-	-
1036	VK-13	+	+	+	-	-	-	-
BP178	KL-29	+	+	+	+	+	+	-
RIJK2	RV-12	+	+	+	-	-	-	-

+: Clear plaque formation; -: no plaque formation.

Supplementary Table S2. The statistical analysis consisted of counting the coloured spots (green: SYTO9 and red: PI) in the different channels of 10 replicas for the two concentrations of each antimicrobial peptide and the relative percentages of viable cells is calculated. The histogram shows the % of cells inhibition.

	250 µM		50 µM	
	**SYTO9**	**PI**	**SYTO9**	**PI**
**KL29**	74	746	0	993
**IL14**	123	132	35	192
**FG22**	228	237	97	253
**GF19**	108	62	90	115
**CONTROL**	325	10		
	**SYTO9**	**PI**		

	% of viability	
	**250 µM**	**50 µM**
**KL29**	9	0
**IL14**	48	15
**FG22**	49	28
**GF19**	64	44
**CONTROL**	97	

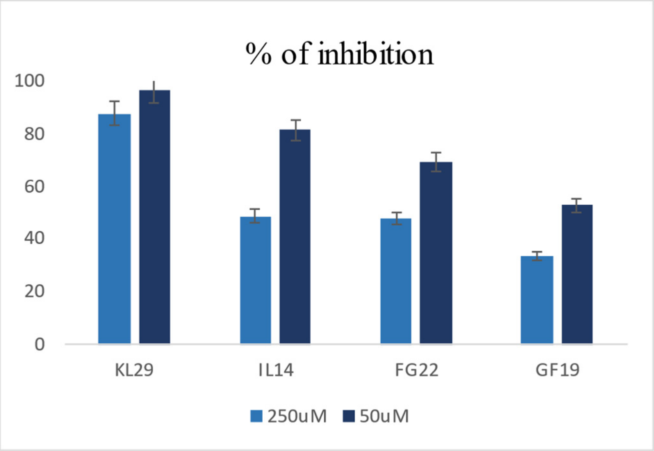

Supplement: Supplementary file 1 [file plants-12-00113-s001.zip › plants-2010124-supplementary.pdf]
